# Serum macrophage migration inhibitory factor reflects adrenal function in the hypothalamo-pituitary-adrenal axis of septic patients: an observational study

**DOI:** 10.1186/1471-2334-9-209

**Published:** 2009-12-21

**Authors:** Takashi Miyauchi, Ryoske Tsuruta, Motoki Fujita, Tadashi Kaneko, Shunji Kasaoka, Tsuyoshi Maekawa

**Affiliations:** 1 Advanced Medical Emergency and Critical Care Center, Yamaguchi University Hospital, 1-1-1 Minamikogushi, Ube, Yamaguchi 755-8505, Japan

## Abstract

**Background:**

The hypothalamo-pituitary-adrenal (HPA) axis modulates the inflammatory response during sepsis. Macrophage migration inhibitory factor (MIF), which counteracts the anti-inflammatory activity of glucocorticoid (GC), is one of the mediators of the development of inflammation. An inflammatory imbalance involving GC and MIF might be the cause or result of adrenal insufficiency. Our objective was to clarify the relationship between serum MIF and adrenal function in the HPA axis of sepsis patients using the adrenocorticotropic hormone (ACTH) stimulation test.

**Methods:**

An observational study was performed in a university intensive care unit over a two-year period. Of 64 consecutive sepsis patients, 41 were enrolled. The enrolled patients underwent an ACTH stimulation test within 24 h of the diagnosis of severe sepsis or septic shock. Clinical and laboratory parameters, including serum MIF and cortisol, were measured.

**Results:**

Based on their responses to the ACTH stimulation test, the patients were divided into a normal adrenal response (NAR) group (n = 22) and an adrenal insufficiency (AI) group (n = 19). The AI group had significantly more septic shock patients and higher prothrombin time ratios, serum MIF, and baseline cortisol than did the NAR group (*P *< 0.05). Serum MIF correlated significantly with the SOFA (Sequential Organ Failure Assessment) score, prothrombin time ratio, and delta max cortisol, which is maximum increment of serum cortisol concentration after ACTH stimulation test (rs = 0.414, 0.355, and -0.49, respectively, *P *< 0.05). Serum MIF also correlated significantly with the delta max cortisol/albumin ratio (rs = -0.501, *P *= 0.001). Receiver operating characteristic curve analysis identified the threshold serum MIF concentration (19.5 ng/mL, *P *= 0.01) that segregated patients into the NAR and AI groups.

**Conclusions:**

The inverse correlation between serum MIF and delta max cortisol or the delta max cortisol/albumin ratio suggests that high serum MIF reflects an insufficient adrenal response in the HPA axis. Serum MIF could be a valuable clinical marker of adrenal insufficiency in sepsis patients.

## Background

Sepsis remains one of the main causes of death in intensive care units, despite enormous clinical efforts to counter it. Although several new interventions for severe sepsis and septic shock have been introduced, few trials have led to a dramatic improvement in mortality [1-7].

It has recently been suggested that decreased adrenal steroid levels during stress (adrenal insufficiency) or tissue resistance to glucocorticoid (GC) are aggravating factors in critically ill patients [8,9]. Adrenal insufficiency, which is commonly diagnosed with the adrenocorticotropic hormone (ACTH) stimulation test, affects the efficacy of GC in the treatment of sepsis and other inflammatory diseases [8,9]. This observation suggests that GC remains an encouraging therapeutic agent for sepsis. Several reports have suggested that GC has beneficial effects [10,11], whereas other studies have indicated that it is not only ineffective, but also harmful [12,13]. To render GC therapy successful in the treatment of sepsis, it is necessary to modulate the inflammatory balance involving GC.

In the present study, we focused on macrophage migration inhibitory factor (MIF) as an indicator of adrenal insufficiency. MIF was first discovered in 1966 as a T-cell cytokine released in the delayed-type hypersensitivity reaction [14]. It has been studied as an immunoneuroendocrine mediator released by various cells, including pituitary cells, macrophages, monocytes, and vascular endothelial cells, in critically ill patients [15,16]. Interestingly, the unique characteristics of MIF specifically counteract GC-induced anti-inflammatory activity, and low concentrations of GC induce MIF production by macrophages [17,18]. These facts suggest that MIF and GC are reciprocally involved in regulating the inflammatory and anti-inflammatory balance [18]. Therefore, serum MIF and GC might be indicators of the inflammatory/anti-inflammatory balance in sepsis patients.

We hypothesized that serum concentrations of MIF are associated with the changes in serum 4 cortisol that occur after ACTH stimulation in sepsis patients.

## Methods

### Patients

**Figure 1:**
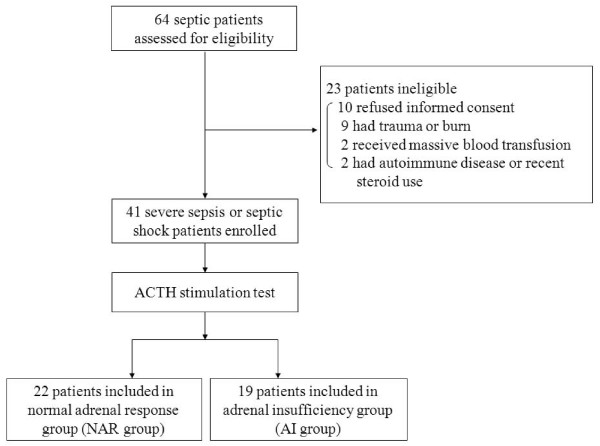
**Study flow diagram**. Sixty-four septic patients were assessed for their eligibility for this study. Of these, 23 were excluded and 41 were enrolled. All the enrolled patients underwent an ACTH stimulation test and were divided into two groups: the normal adrenal response group (NAR group) and the adrenal insufficiency group (AI group).

This study was approved by our Institutional Review Board in Yamaguchi University Hospital. We obtained the informed consent of the patients' families or next of kin at the time of enrolment. Patients admitted between May 2006 and May 2008, and who met the criteria for severe sepsis or septic shock, were prospectively assessed [19]. Figure 1 shows the selection of the patients. Sixty-four patients were assessed for eligibility for this study, and 23 patients were excluded. The ACTH stimulation test was performed in the 41 enrolled patients, of whom 26 had severe sepsis and 15 had septic shock. The sites of infection are listed in Table 1, as determined based on clinical manifestations, positive culture samples, X-ray films, or computed tomographic imaging.

**Table 1:** Comparison of the clinical and laboratory characteristics of the normal adrenal response group and the adrenal insufficiency group of patients

	NAR group (n = 22)	AI group (n = 19)	*P *value
Age (yrs)	67 (50-71)	68 (55-74)	0.574
Male (n)	14	16	0.233
APACHE II	27 (21-29)	27 (19-32)	0.582
SOFA score	9 (5-11)	8 (6-11)	0.793
JAAM DIC score	3.0 (1.75-5.0)	3.0 (2.0-5.0)	0.433
Severe sepsis/septic shock (n)	19/3	7/12	0.001
Survivors/non-survivors (n)	16/6	13/6	0.763
Albumin (g/dL)	2.1 (1.7-2.5)	2.0 (1.6-2.2)	0.636
Prothrombin time ratio	1.2 (1.1-1.3)	1.3 (1.2-1.5)	0.025
MIF (ng/mL)	16.6 (14.5-20.1)	23.8 (17.6-44.5)	0.010
Baseline cortisol (μg/dL)	18.9 (14.3-25.3)	27.6 (17.6-46.8)	0.028
Baseline cortisol/albumin ratio	10.3 (8.6-13.0)	15.1 (9.4-20.0)	0.053
Delta max cortisol (μg/dL)	15.5 (9.9-19.6)	4.6 (1.4-7.3)	< 0.001
Delta max cortisol/albumin ratio	7.5 (6.0-8.5)	2.3 (0.8-3.3)	< 0.001
			
Site of infection (n)			
Respiratory tract	11	7	
Skin and soft tissue	3	5	
Gastrointestinal tract	2	3	
Urinary tract	1	2	
Peritonitis	2	1	
Meningitis	1	0	
Infectious endocarditis	1	0	
Unknown	0	1	

All values are presented as medians (interquartile ranges).

NAR: normal adrenal response; AI: adrenal insufficiency; APACHE II: Acute Physiology and Chronic Health Evaluation II; SOFA score: Sequential Organ Failure Assessment score; JAAM DIC score: Disseminated Intravascular Coagulation score established by the Japanese Association for Acute Medicine; Prothrombin time ratio: the ratio of the actual prothrombin time vs the normal control prothrombin time; MIF: macrophage migration inhibitory factor; Delta max cortisol: maximum increment in the cortisol concentration caused by the adrenocorticotropic hormone stimulation test.

### ACTH stimulation test and patient classification

The ACTH stimulation test was performed in all enrolled patients within 24 h of the diagnosis of severe sepsis or septic shock. Synthetic ACTH (tetracosactide acetate, 0.25 mg) was administered intravenously, and serum cortisol concentrations were measured before (baseline) and 30 and 60 min after ACTH administration. The maximum increment in the serum cortisol concentration is designated the "delta max cortisol." To estimate the level of free cortisol, a normalized value for serum albumin (cortisol/albumin ratio) was used as an index of free cortisol.

Patients were divided into two groups: the normal adrenal response group (NAR group; delta max cortisol ≥ 9 μg/dL and random baseline ≥ 10 μg/dL) and the adrenal insufficiency cortisol group (AI group; delta max cortisol < 9 μg/dL or random baseline cortisol < 10 μg/dL), based on previous reports (Figure 1) [8,9,20-22]. None of the patients presented a random baseline cortisol concentration below 10 μg/dL.

### Blood sampling, measurement of serum MIF and cortisol, and other laboratory data

Arterial blood samples were centrifuged at 3,000 × g or 10 min after incubation for at least 30 min at 4°C. The supernatant (serum) was stored in sterile tubes and frozen at -80°C. Serum MIF concentrations were measured using an enzyme-linked immunosorbent assays (ELISA) (human MIF ELISA kit, Sapporo I.D.L., Japan). The serum sample and an enzyme-labelled antibody were injected onto a solid phase (ELISA plate) that contained a mouse monoclonal antibody, so that the serum MIF was sandwiched between the mouse monoclonal antibody and the enzyme-labelled antibody. The plate was cleaned with washing solution to remove any unbound antibody. The bound enzyme-labelled antibody was subjected to a colorimetric reaction and the colour intensity was measured at 450 nm.

The serum cortisol concentrations were measured with an enzyme immunoassay (ST AIA-PACK CORT, TOSOH-AIA, Japan). The cortisol in the test sample competed with the enzyme-labelled cortisol for a limited number of binding sites on a cortisol-specific antibody, which was immobilized on magnetic beads. The beads were washed to remove the unbound enzyme-labelled cortisol and then incubated with the fluorogenic substrate 4-methylumbelliferyl phosphate. The amount of enzyme-labelled cortisol that bound to the beads was inversely proportional to the cortisol concentration in the test sample. A standard curve, based on a range of known concentrations, was constructed and the unknown cortisol concentrations were calculated using this curve. The measured cortisol value is a total one.

### Statistical analysis

The data are presented as medians and interquartile ranges. Comparisons between the NAR 6 group and the AI group were made using the Mann-Whitney U test and the χ^2 ^test. The correlations between the serum MIF concentrations and the other clinical and laboratory parameters were analyzed with Spearman's correlation coefficient (rs). Receiver operating characteristic (ROC) curve analysis was used to determine the threshold of MIF that allowed the segregation of the patients into different groups. All analyses were performed with the SPSS software, version 11.01 (SPSS, Chicago, IL, USA). Two-sided *P *values of < 0.05 were considered significant.

## Results

### Comparison of the NAR and AI groups

The enrolled patients were divided into two groups: the NAR group (n = 22) and the AI group (n = 19), based on the results of the ACTH stimulation test (Table 1). The number of septic shock patients was significantly greater in the AI group than in the NAR group. Mortality was not significantly different between the two groups. None of the clinical and laboratory data, with the exception of the prothrombin time ratio, were significantly different between the two groups. There was also no difference between the two groups regarding the site of infection.

Table 1 presents a comparison of serum MIF, baseline cortisol, and the results of the ACTH stimulation test between the two groups. Serum MIF was significantly higher in the AI group than in the NAR group. Baseline cortisol was significantly higher in the AI group than in the NAR group, and the baseline cortisol/albumin ratio, which was used as an index of baseline free cortisol, was relatively but not significantly higher in the AI group. Delta max cortisol was significantly lower in the AI group, and the delta max cortisol/albumin ratio was significantly lower in the AI group than in the NAR group.

### Correlations between serum MIF and clinical and laboratory parameters

Table 2 lists the correlations between the serum MIF concentrations and the clinical and laboratory parameters. Serum MIF correlated inversely with delta max cortisol and the delta max cortisol/albumin ratio. The Sequential Organ Failure Assessment (SOFA) score and prothrombin time ratio also correlated significantly with the serum MIF concentration. Serum MIF showed no correlation with baseline cortisol or the baseline free cortisol index.

**Table 2:** Correlations between serum macrophage migration inhibitory factor and other clinical and laboratory parameters

	rs	*P *value
APACHE II	0.144	0.371
SOFA score	0.414	0.007
JAAM DIC score	0.267	0.092
Albumin	-0.290	0.860
Prothrombin time ratio	0.355	0.024
Baseline cortisol	0.212	0.182
Baseline cortisol/albumin ratio	0.237	0.135
Delta max cortisol	-0.490	0.001
Delta max cortisol/albumin ratio	-0.501	0.001

rs: Spearman's correlation coefficient; APACHE II: Acute Physiology and Chronic Health Evaluation II; SOFA score: Sequential Organ Failure Assessment score; JAAM DIC score: Disseminated Intravascular Coagulation score established by the Japanese Association for Acute Medicine; Prothrombin time ratio: the ratio of the actual prothrombin time vs the normal control prothrombin time; Delta max cortisol: maximum increment in the cortisol concentration caused by the adrenocorticotropic hormone stimulation test.

### ROC curve analysis

**Figure 2:**
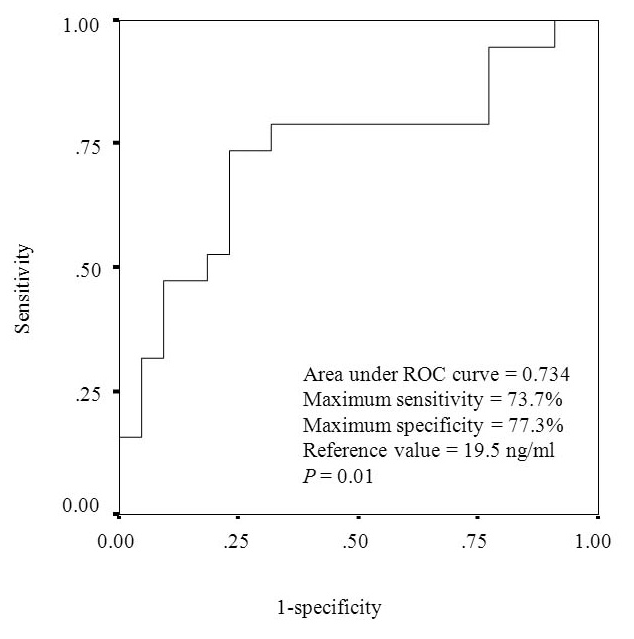
**ROC curve of serum MIF concentration used to segregate patients into the NAR and AI group**. The area under the curve was 0.734, and the maximum sensitivity and specificity were 0.737 and 0.773, respectively. The reference value for the segregation of patients into the two groups was 19.5 ng/mL (*P *= 0.01).

An ROC curve analysis was conducted to determine the threshold serum MIF concentration that segregated the patients into the NAR and AI groups (Figure 2). The area under the ROC curve was 0.734 and the MIF concentration value with the highest sensitivity and specificity was the reference value of 19.5 ng/mL, with a sensitivity of 0.737 and specificity of 0.773 (*P *= 0.01).

## Discussion

MIF is secreted by monocytes/macrophages after infectious stimulation and acts as a pro-inflammatory cytokine. An increase in MIF in critically ill patients and a correlation between serum MIF and early death in patients with severe sepsis have previously been reported [23-26]. It is noteworthy that MIF counteracts GC-induced anti-inflammatory activity and that low concentrations of GC induce MIF production by macrophages [17,18]. We speculated that the serum MIF concentration might reflect adrenal function in the hypothalamo-pituitary-adrenal (HPA) axis in patients with sepsis.

The main and novel result of this study is the inverse correlation between the serum MIF concentration and delta max cortisol (Table 2). A correlation between serum MIF and total baseline cortisol was reported previously [26,27]. However, this is the first report to suggest an association between MIF and the adrenal response. In general, free cortisol is preferable to total cortisol in evaluating the adrenal function of the HPA axis, especially during excessive inflammation [28]. Free cortisol has physiological effects, but the direct measurement of free cortisol is complicated. Therefore, the ratio of total cortisol/albumin has been used as an index of free cortisol when evaluating HPA axis function [29,30]. Changes in the free cortisol index reflect the status of the inflammatory balance regulated by the HPA axis. In this study, a significant inverse correlation between serum MIF and the delta max cortisol/albumin ratio was observed (Table 2). Consequently, high serum MIF is considered to be associated with insufficient activation of the HPA axis in patients with severe sepsis or septic shock.

The dose of synthetic ACTH (0.25 mg) used in the present study was about 100-fold higher than the ACTH levels observed under physiological stress. Therefore, the serum cortisol concentration might reach its maximum level after the ACTH stimulation test. This means that the delta max cortisol value reflects the reserve capacity of the adrenal gland. A low delta max cortisol suggests that the patient's adrenal glands are already highly activated and that his/her cortisol production isare insufficient to cope with the added stress. Therefore, the baseline cortisol observed in the AI group could be insufficient to accommodate serious stress, even though it was significantly higher than that in the NAR group (Table 1). Therefore, the inverse correlation between serum MIF and delta max cortisol indicates that a high concentration of serum MIF might be associated with a relative insufficiency of innate cortisol activity or HPA axis dysfunction (Table 2).

The secretion of cortisol and the translocation of the GC receptor complex into the nucleus are impaired in critically ill patients [31]. The pathophysiological condition of reduced steroid production (adrenal insufficiency) or tissue resistance to GC activity during critical illness is called "critical -illness-related corticosteroid insufficiency" (CIRCI) [22]. CIRCI is defined as inadequate cellular corticosteroid activity for the additional stress experienced by the patient. Although the baseline cortisol concentration and baseline cortisol/albumin ratio in the AI group were higher than those in the NAR group (Table 1), they were not high enough to suppress the inflammatory response in the AI group patients. As a result, we could determine the threshold serum MIF level that defines adrenal insufficiency. Using ROC curve analysis, we demonstrated that the threshold serum MIF level that segregated patients into the NAR group and AI group was 19.5 ng/mL and that the maximum sensitivity and specificity of this measure were 0.737 and 0.773, respectively (Figure 2). These results suggest that serum MIF is a good clinical marker of adrenal insufficiency.

In the present study, the SOFA score correlated significantly with serum MIF (Table 2). This result is consistent with previous reports that serum MIF is related to the severity of sepsis and mortality [23-26]. Macrophages contain a certain amount of pre-formed MIF, which is rapidly released with infectious stimulation [15]. MIF secretion from macrophages also occurred under challenge with low concentrations of lipopolysaccharide, which were 10- to 100-fold lower than the concentration necessary to induce tumor necrosis factor α. MIF induced cytokine release, neutrophil accumulation, and persistent inflammation [15,32]. It has also been reported that the administration of recombinant MIF increased the systemic toxicity of endotoxin, whereas anti-MIF antibody fully protected mice from endotoxic shock and death [17]. Consequently, we consider that the measurement of the serum MIF concentration in the early phase of sepsis is useful in detecting and even treating inflammatory imbalance and organ failure.

The correlation between serum MIF and the prothrombin time ratio indicates that an increase in the prothrombin time ratio predicts sequential organ failure. Moreover, the increase in serum MIF may indicate coagulopathic dysfunction. MIF has recently become both an attractive marker for prognostic prediction and a therapeutic target, because levels of MIF are associated with the mechanisms of both inflammation and coagulation in sepsis [33]. The main target of sepsis treatment is inflammatory modulation, in which the activation of the HPA axis is involved. The relationship between MIF and delta max cortisol in the HPA axis network has been demonstrated in 10 the present study.

Because the measurement of serum MIF concentrations is not complicated, it is feasible during routine laboratory practice. Unlike the ACTH stimulation test, we can repeatedly measure the MIF concentration within a short time period. Daily monitoring might be useful in evaluating the inflammation status and adrenal responses of critically ill patients.

There are several limitations to this study. First, additional information is required to clarify the detailed mechanism involving adrenal function, the HPA axis, and MIF in critically ill patients. Second, we used the cortisol/albumin ratio as the index of free cortisol to evaluate the adrenal function in the HPA axis. Although measurement of the free cortisol value is preferable, this procedure is not currently widely available. However, it is probable that with improvements in laboratory techniques and increased clinical demand, the method will become commercially available [34]. Third, the number of patients enrolled in this study was relatively small. We intend to continue this study of the relationship between MIF and the HPA axis.

## Conclusions

An inverse correlation was demonstrated between MIF and delta max cortisol or the delta max cortisol/albumin ratio in patients with severe sepsis or septic shock. MIF may play an important role in sepsis as a pro-inflammatory cytokine and an inflammatory modulator, and may be a marker of the severity of the associated adrenal insufficiency.

## List of abbreviations

GC: glucocorticoid; ACTH: adrenocorticotropic hormone; MIF: macrophage migration inhibitory factor; NAR group: normal adrenal response group; AI group: adrenal insufficiency group; ELISA: enzyme-linked immunosorbent assay; ROC: receiver operating characteristic; SOFA: Sequential Organ Failure Assessment; HPA axis: hypothalamo-pituitary-adrenal axis; CIRCI: critical-illness-related corticosteroid insufficiency.

## Competing interests

The authors declare that they have no competing interests.

## Authors' contributions

TM, RT, and TM designed the study. TM, MF, and TK collected the data. TM performed the statistical analyses and wrote the manuscript, and all authors participated in its critical revision. TM had the final responsibility for the decision to submit the manuscript for publication. All authors read and approved the final manuscript.

## Pre-publication history

The pre-publication history for this paper can be accessed here:

http://www.biomedcentral.com/1471-2334/9/209/prepub
